# Effects of Parathyroid Hormone on Immune Function

**DOI:** 10.1155/2010/418695

**Published:** 2010-09-16

**Authors:** Abdallah Sassine Geara, Mario R. Castellanos, Claude Bassil, Georgia Schuller-Levis, Eunkue Park, Marianne Smith, Michael Goldman, Suzanne Elsayegh

**Affiliations:** ^1^Department of Medicine, Staten Island University Hospital, 475 Seaview Avenue, Staten Island, NY 10305, USA; ^2^NYS Institute for Basic Research in Developmental Disabilities, 1050 Forest Hill Road, Staten Island, NY 10314, USA; ^3^Division of Nephrology, Staten Island University Hospital, 475 Seaview Avenue, Staten Island, NY 10305, USA

## Abstract

Parathyroid hormone (PTH) function as immunologic mediator has become interesting with the recent usage of PTH analogue (teriparatide) in the management of osteoporosis. Since the early 1980s, PTH receptors were found on most immunologic cells (neutrophils, B and T cells). The in vitro evaluations for a possible role of PTH as immunomodulator have shown inconsistent results mainly due to methodological heterogeneity of these studies: it used different PTH formulations (rat, bovine, and human), at different dosages and different incubating periods. In some of these studies, the lymphocytes were collected from uremic patients or animals, which renders the interpretation of the results problematic due to the effect of uremic toxins. Parathyroidectomy has been found to reverse the immunologic defect in patients with high PTH levels. Nonetheless, the clinical significance of these findings is unclear. Further studies are needed to define if PTH does have immunomodulatory effects.

## 1. Introduction

Infection remains a major cause of morbidity and mortality in patients with end-stage renal disease (ESRD). Hospitalization rates for infections have risen since 1993, 19% for pneumonia, 24% for cellulitis, and 29% for sepsis/bacteremia. Death from sepsis is 50 times higher in hemodialysis patients than in the general population even after accounting for other comorbidities [1, 2]. Several factors make patients with ESRD susceptible to infections; one of the most difficult causes to treat is the development of an acquired immune dysfunction associated with chronic kidney disease (CKD) and dialysis therapy [3–5]. The first evidence for this dysfunction came from early observations which showed that patients attending dialysis units had endemic outbreaks of hepatitis B [6]. In addition, the clinical course for dialysis patients that acquired hepatitis B was worse; 60% of these patients became chronic carriers as compared to only 5% in the general population. Studies of vaccine efficacy corroborated these findings and helped to elucidate the pathogenic mechanism behind the immune defect. When patients with ESRD were vaccinated with protein-based vaccines, such as hepatitis B [7], influenza virus [8], and clostridium tetani [9], which require a T-lymphocyte-dependent response, there were high failure rates. In contrast, effective immunity was achieved using polysaccharide pneumococcal vaccine since this vaccine activates directly B cells without interaction with T-lymphocyte [10]. These observations and subsequent studies support the concept of an acquired T-lymphocyte dysfunction in ESRD patients. Though patients with ESRD are lymphopenic compared to healthy subjects, this effect is slight and would not explain the significant immune defect seen in this population [11]. 

In vitro studies examining T-lymphocyte function during exposure to various mitogens demonstrate a significant impairment in T-lymphocyte proliferation [12] and a reduction in activation-dependent cytokine production (interleukin-2,6,10, *α*-interferon, and tumor necrosis factor alpha) [13]. This decrease in T-lymphocyte function was further identified to be an impairment in the interaction between antigen-presenting cells (APCs) and T-lymphocytes [3]. Therefore, although patients with ESRD have functionally normal T and B lymphocyte, the T and B lymphocytes do not respond appropriately, as they are not receiving normal signals from APCs. This impairment occurs via two mechanisms [3, 14]. Firstly, uremia causes a reduction of the essential costimulatory molecule B7-2 (CD86) on antigen-presenting cells which decreases the activation of helper T-lymphocyte cells [15]. This effect can be improved with dialysis therapy. Secondly, however, hemodialysis itself causes activation of antigen-presenting cells [14], which in addition to causing an immunity impairment, is now also believed to be responsible for the malnutrition-inflammation-atherosclerosis syndrome seen in patients with ESRD [16]. Similarly, a polymorphonuclear leukocyte (PMNL) dysfunction has been observed in dialysis patients with impairment in migration, chemotaxis, and reduced ability to kill intracellular micro-organisms [17]. 

Over the last 3 decades, secondary hyperparathyroidism in ESRD patients has been studied as a possible factor in the development of an acquired immune dysfunction [18]. PTH is an 84-amino acid polypeptide secreted by the parathyroid gland. Its homeostasis is mainly controlled by calcium/phosphorus regulation. High levels of PTH have been implicated in several complications associated with uremia. PTH adversely affects the metabolism of various cells and organs; it causes osteitis fibrosa cystica, cardiac arrhythmias [19, 20], peripheral neuropathy [21], anemia (by inhibiting precursors of erythropoiesis) [22, 23], and glucose intolerance [24, 25].

Kaplan et al. in the early 1970s [26] reported an increase in malignant neoplasms discovered at autopsy in patients with primary hyperparathyroidism. Similarly, there have been reports of an increase in the incidence of leukemia [27] and monoclonal gammopathy [28] in patients with primary hyperparathyroidism. Though these observations could not be definitively linked to excess PTH, the concept that PTH may modulate the immune response was further supported by Perry et al. [29] in describing a receptor for PTH in circulating human lymphocytes. While the immune defect in CKD appears to be multifactorial, the contribution of PTH, if any, remains unclear. This potential immune impairment of PTH is clinically relevant, since in theory a parathyroidectomy or medical treatment may reverse this effect. We present a narrative review of the literature relevant to PTH as an immune modulator and examine the effects of hyperparathyroidism on immune function in patients with CKD.

## 2. Parathyroid Hormone Receptors and Leukocytes

The observation that several immunodeficiency syndromes had associated bone abnormalities has led to the search for a link between leukocytes and bone cells (osteoblasts and osteoclasts). Various cytokines have osteoclasts as target sites [30–34]. These cytokines affected bone remodeling by activating a PTH receptor on the monocyte-like precursor of osteoclasts [30]. Yamamoto et al. [35] were the first to identify the presence of PTH receptors on bovine lymphocytes. Perry et al. [29] demonstrated PTH receptors on human mononuclear cells and subsequently identified receptors on additional leukocytes [36]. The mechanism by which PTH influences leukocytes is not well understood, yet there appears to be an increase in intracellular calcium level. Potentially, this might lead to an increase in cellular adenylate cyclase activity [29, 35]

## 3. Parathyroid Hormone and T Lymphocytes (Table 1)

Studies examining the effect of PTH on T lymphocytes are neither consistent nor conclusive (see Table 1). Most studies showed that PTH produces an inhibitory effect on various parameters of the immune system [37–39], while other studies demonstrated that PTH had a stimulatory function under certain laboratory conditions [40, 41].

Shasha et al. [37] were one of the first to show that when peripheral blood lymphocytes from healthy donors were incubated with increasing concentrations of PTH, a marked inhibition of E rosette formation was produced. Massry et al. [46] found a direct correlation between PTH levels in uremic patients and the degree of inhibition of lymphocyte proliferation. More recently, Kaneko et al. [39] found an decrease in T-lymphocyte proliferation from patients with ESRD when recombinant PTH (rPTH) was added in vitro and that the decrease in proliferation was dose-dependent. In the control group, which consisted of T lymphocytes isolated from healthy adults, proliferation was increased by addition of rPTH. This stimulatory effect on proliferation was reversed and inhibited by adding urea or an acid (pH = 7.0). The authors concluded that the T-lymphocyte response to PTH is modulated by the uremic state.

In examining the other human studies that found a stimulatory effect of PTH on leukocytes obtained from healthy donor volunteers [37], the PTH used was bovine (not human), and the lymphocytes were incubated for longer periods (5 days). These differences may account for the inconsistent study results, making it difficult to determine if PTH affects the immune system in the clinical setting. 

In addition, the results of in vitro assays are difficult to correlate to patients with CKD. Doses of hormones in laboratory studies use concentration ranges only as low as 2 to 1.6 *μ*M, while clinically, PTH is measured in pg/ml. Even in ESRD patients that develop severe tertiary hyperparathyroidism with PTH levels that may rise above 1000 pg/ml to levels in the upper range of 3000 pg/ml (about 315 picomoles) [46], serum PTH levels are still several fold lower than the concentration used in the *in vitro* studies. 

Overall, results from laboratory assays alone are difficult to interpret, especially since they only provide some insight into the effects of acute exposure to PTH. Chronic exposure to PTH may affect immune cells differently; thus *in vitro* data needs to be evaluated with caution. Our laboratory recently completed a study evaluating the effects of PTH on the production of IL-6 and IL-8 from activated leukocytes from healthy donor volunteers (work still in press). Our results demonstrated no effect of PTH on the production of IL-6 and IL-8 and that leukocyte proliferation was inhibited only at the highest dose of PTH (0.8 *μ*M) tested. 

Few studies exist that attempt to categorize changes of peripheral blood leukocytes in CKD patients. One described a slight lymphopenia compared to the leukocyte count in normal subjects [47–49]. Another studied the effect of PTH on the distribution of T-lymphocytes subpopulations (CD4 lymphocytes, CD8 lymphocytes, and CD4/CD8 ratio) (see Table 2) [42–45]. Ozdemir et al. [45] found that in ESRD patients the CD4/CD8 lymphocyte ratio was increased in the presence of high serum PTH levels. In contrast, Angelini et al. [42] studied patients with ESRD and found that patients with elevated PTH had a decrease in CD4, an increase in CD8 lymphocytes, and thus a decrease in the CD4/CD8 lymphocyte ratio. There was a linear correlation between the levels of PTH and CD8 lymphocytes and a reverse correlation between level of PTH and total T-lymphocytes, CD4 lymphocytes, and CD4/CD8 ratio. Klinger et al [40] found that (1–84) PTH stimulated proliferation of T lymphocytes in a dose-dependent manner, and that the hormone did not alter the CD4/CD8 ratio. Inactivation of PTH cancelled this stimulatory effect.

## 4. Restoration of T-Lymphocytes Function: Parathyroidectomy and Calcium Channel Blockers (Table 2)

Giacchino et al. [54] reported that the inhibitory capacity of serum taken from uremic patients on E rosette formation was decreased following parathyroidectomy. Shasha et al. [50] examined T-cell function in primary hyperparathyroidism both before and 1 month after parathyroidectomy. The hyperparathyroid patients, prior to surgery, demonstrated low total T-lymphocytes count, increased CD8 lymphocytes, decreased CD4/CD8 ratios, and a decreased ability of T-lymphocytes to become activated in comparison to healthy controls. All of these abnormalities were restored following parathyroidectomy. Similarly, Kotzmann et al. [53] reported a restoration of lymphocyte responsiveness to stimulation 6 months after parathyroidectomy in patients with primary hyperparathyroidism. Tzanno-Martins et al. [44] studied the consequence of parathyroidectomy in hemodialysis patients and showed that patients with extremely high levels of PTH had a complete restoration of impaired T-lymphocyte proliferation after parathyroidectomy.

## 5. Parathyroid Hormone and B Lymphocytes

Although the initial evidence found during the studies of vaccination in CKD was more suggestive of an indirect effect of PTH on B lymphocytes via T-lymphocytes dysfunction, the discovery of PTH receptors on B lymphocytes has favored a more direct effect of PTH. This has been found in several clinical experiments, where PTH was found to affect several aspects of the B-cell function (proliferation, antibodies production, and metabolism) (Table 3).

Alexiewicz et al. [38] found that both the intact molecule of (1–84) PTH and its amino-terminal fragment (1–34) PTH caused dose-dependent inhibition of B-lymphocyte proliferation in normal subjects. After the activity of the amino-terminal region was inhibited through oxidation of the SH residues, the inhibitory effect was preserved, suggesting that the mechanism of PTH is mostly mediated through the carboxyl-terminal region. This inhibitory influence is most likely mediated by the stimulation of cyclic AMP production. Furthermore, Gaciong et al. [51, 56] reported that the defect in antibody production in uremic patients was due to the direct action of PTH on B lymphocytes, and that B lymphocytes from ESRD patients produce very low amounts of IgG following T-cell stimulation in vitro. 

Clinically, the plasma levels of IgG, IgM, and IgA are usually in the normal range in uremic patients, while specific antibody responses are significantly depressed [57, 58]. The response to vaccination against hepatitis B was used as a sensitive clinical marker for B-cell dysfunction [7]. Deficient reaction to vaccination was also documented for influenza [8], tetanus [9], and diphtheria. Pneumococcal vaccination is an exception, as uremic patients responded with normal antibody titers to each antigen type [10]. Since B lymphocytes recognition of polysaccharide antigens is a T-cell independent interaction, vaccination using polysaccharide antigens in the pneumococcal vaccine was sufficient. This implies that the defect in antibody production was more due to T-cell-B-cell interaction. 

Since PTH function on B-lymphocyte cells was mediated through alteration of intracellular calcium metabolism, by using the calcium channel blocker nifedipine, Alexiewicz et al. [59, 60] were able to reverse the abnormalities of intracellular Ca concentration and restore adequate proliferation of B cells following stimulation.

## 6. PTH and Polymorphonuclear Leukocytes

PMNLs of patients with elevated PTH serum levels presented impaired migration [61], reduced phagocytic [62] and bactericidal activity [63], and an inhibited chemotaxis [64]. 

The first observations were found in patients with primary hyperparathyroidism and normal renal function. The migration and chemotaxis of PMNLs were impaired, however these abnormalities disappear after parathyroidectomy [64]. Tuma et al. [65] demonstrated that secondary hyperparathyroidism is either directly or indirectly responsible for the altered leukocyte function in patients with uremia, particularly those with marked elevation of PTH. Massry et al. [66] verified that (1–84) PTH stimulated elastase release from PMNL in a dose-dependent and time-dependent manner. This effect was mostly mediated by the carboxyl-terminal region of the hormone (1–34), and PTH had no stimulatory effect on elastase release. In addition, Doherty et al. [61] demonstrated that migration of PMNL from patients with advanced renal failure was reduced and that there is an inverse relationship between random migration of PMNLs and serum levels of PTH. Esposito et al. [67] studied the role of PTH in depressing PMNLs phagocytic function in uremia. The data produced shows that phagocytosis is lowered in uremic patients with both low and high plasma PTH but more noticeably in the group with high plasma PTH. Similarly, the contact angle of cells during phagocytosis was affected more in patients with high PTH levels.

The mechanism through which PTH impaired PMNL function is multifactorial. Alexiewicz et al. [62] found that chronic exposure to excess PTH may cause accumulation of calcium in PMNLs that leads to derangement of the intracellular cascade, which occurs during the process of phagocytosis (especially after the Fc gamma RIII receptors of PMNLs interact with antibody fixing the antigen). Horl et al. [68] found that in ESRD patients, PTH not only elevated basal levels of cytosolic calcium, it also altered carbohydrate metabolism. Glucose uptake, glycogen synthetase activity, and glycogen content were all reduced. Kiersztejn et al. [69] also found that both basal and stimulated O2 consumption of PMNLs in CKD subjects and rats was lower than normal.

Since increased intracellular calcium is the principal mechanism in PMNL dysfunction, calcium channel blocker therapy has been tested with some success. Studies in CKD rats demonstrate that these derangements in PMNLs could be prevented by a previous parathyroidectomy or treatment with verapamil. In ESRD patients, eight to nine weeks of verapamil therapy (120 mg/day) normalized the elevated intracellular calcium concentration and carbohydrate metabolism in PMNLs. However, these beneficial effects were lost after eight to ten weeks of discontinuation of verapamil treatment [68]. 

Alexiewicz et al. [60] found that treatment with nifedipine was associated with the return of intracellular calcium concentration toward normal values; it also restored the ATP content of PMNLs. The normalization of intracellular calcium concentration and restoration of PMNL dysfunction by long-term therapy with calcium channels blockers were also demonstrated in diabetic patients treated with amlodipine [70]. Five months after stopping nifedipine treatment, the positive activity of calcium channel blockers on phagocytosis was lost [61]. Haag-Weber et al. [71] found that continuous infusion of nifedipine in a dose of 18 micrograms/kg/h during HD completely inhibited the rise of cytosolic free calcium during dialysis. PMNL dysfunction could be reversed by parathyroidectomy [65, 69].

## 7. Explanation of the Discrepancy between In Vitro and In Vivo Studies

Although the current published studies demonstrate that PTH can influence various parameters of the immune system in both normal and ESRD subjects, the results of the laboratory studies are inconsistent, and their clinical significance still needs further investigation. What prevents a more definitive conclusion to be made when examining and comparing these in vitro studies is that the dosages of PTH used in these laboratory studies are much higher than the physiological ranges of PTH. Even compared to the abnormally high ranges seen in ESRD patients with tertiary hyperparathyroidism, the doses used in laboratory experiments were several logs higher. However, the effect of this higher concentration is decreased by the fact that leukocytes counts in wells during in vitro studies are also several log higher than blood leukocytes in patients. Therefore, the ratio of PTH to leukocytes may be more important to reflect in vivo conditions. Furthermore, chronic exposure that occurs in CKD is difficult to study in vitro, since human leukocytes cultures are short term (<5 days). A possible model for studying chronic exposure of PTH in laboratory experiments would be to use cell lines from immortalized human leukocytes. However, no good laboratory model has been developed for examining chronic exposure to PTH in leukocytes to date. Another difficulty in experimental studies was that various sources of PTH were used (rat, bovine, and human) and varying conditions of culture. Although there are limitations and difficulties in interpreting these studies, it remains that PTH receptors are located on leukocytes, and their exact role and function need to be defined. One possible way to determine if hyperparathyroidism associated with ESRD affects immune function is to examine if PTH receptors have a physiologic function in health. This approach seems more reasonable than starting with ESRD patients who already have various metabolic alterations.

## 8. Conclusion

Many of the infectious complications experienced by patients undergoing hemodialysis can be attributed to altered host defenses. Both humoral and cellular-mediated immunities are affected in ESRD patients. Alterations of the immune system in ESRD patients are multifactorial. The evidence presented shows that PTH may be an important factor in influencing this dysfunction. Massry and Fadda. [72] stated in his review that chronic renal failure is a “state of cellular calcium toxicity.” PTH is an essential hormone that initiates the cascade of events leading to increased calcium influx intracellularly, a pivotal step in modulating leukocyte enzymes and biochemical processes.

In conclusion, we present a review of the literature evaluating PTH and immune function. After observing that PTH receptors are located on various immune cells and evaluating the studies that attempt to understand the role of PTH in disease, it is clear that a basic understanding of the normal physiology of the PTH receptor on leukocytes is imperative. Finally, evidence of PTH affecting immunity may not be solely achieved by laboratory studies. Epidemiologic and observational studies should be done from large, maintained databases of ESRD patients, such as the US Renal Data System. Examination of infectious disease death rates and cancer rates can be correlated to PTH level. While the data from the reviewed studies supports the possibility that PTH affects the immune system, further research is needed, particularly since this abnormality could be reversed with the treatment of hyperparathyroidism.

## Figures and Tables

**Table 1:** Study discussing the effect of PTH on T-lymphocytes.

	Study design	PTH	Assays	Results
Shasha et al. [37]	T lymphocytes from 9 healthy subjects	Human and bovine (1–84) PTH.	Stimulation with: Phytohemagglutinin, ConA.	Both PTH had a dose-dependant inhibition of transformation of T-lymphocytes.
	Incubated for 72 hours	60, 300, and 1200 mIU/ml.	Proliferation assessed with thymidine uptake.	Human PTH decreased CD4/CD8 ratio. Both PTH had inhibition E rosette formation.

Alexiewicz et al. [38]	Mononuclear cells from: 33 hemodialysis patients 38 healthy subjects	(1–84) PTH bovine.	Stimulation with: Phytohemagglutinin	T lymphocytes of ESRD patients had lower ability to proliferate and produce IL-2 after stimulation. Adding IL-2 to T-lymphocytes of dialysis patients did no reverse the proliferation defect.
	Cultured 5 days.	4 × 10^−7^ M	Proliferation assessed with thymidine uptake.	

Klinger et al. [40]	T lymphocytes from 34 healthy subjects	Bovine (1–34) PTH, bovine (1–84) PTH.	Stimulation with: Phytohemagglutinin, pokeweed mitogen.	(1–84) PTH increased lymphocytes proliferation in dose-dependent manner, increased IL-2 production. (1–34) PTH increased lymphocytes proliferation but lesser than (1–84) PTH. (1–84) PTH had no effect on CD4/CD8 ratio. Both PTH stimulated cAMP production.
	Cultured 5d.	10^−7^, 2 × 10^−7^ and 4 × 10^−7^ M.	Proliferation assessed with thymidine uptake.	

Lewin et al. [41]	T-lymphocytes from rats: 18 nephrectomy v/s 17 healthy. Then 6/18 and 9/17 had parathyroidectomy. 72 hours incubation.	Rat (1–84) PTH	Stimulation with: Phytohemagglutinin.	T-lymphocyte response to stimulation was higher in uremic rats. Parathyroidectomy reduced T-cell response to PTH which stimulation enhanced T-cell stimulation (dose dependent) only in uremic rats.
		10^−7^, 2 × 10^−7^ and 4 × 10^−7^ M.	Proliferation assessed with thymidine uptake.	
Angelini et al. [42]	54 patient with ESRD: 26 normal serum PTH. 28 high serum PTH. Controls are heathy subjects.	PTH measured with radioimmunoassay.	N/A	Both groups had decreased T-lymphocytes number. Decreased in total CD4 cells number and CD4/CD8 ratio in the group of high serum PTH. Higher total CD8 number in patient with normal serum PTH. PTH shows a linear correlation with CD8 cells and reverse correlation with total T lymphocytes, CD4, and CD4/CD8 ratio.
Angelini et al. [43]	Population of hemodialysis patients: 26 normal PTH, 28 elevated PTH	PTH measured by immunoradiometry	N/A	Both groups have decreased total number of lymphocytes. Reverse correlation between levels of PTH and number of T-lymphocytes. linear correlation between levels of PTH and IL-2 Linear correlation between CD8 cells and PTH.

Kaneko et al. [39]	T lymphocytes from 16 hemodialysis patients	Recombinant human (1–84) PTH.	Stimulation with: anti-CD3 antibody, PPD and allo- antigens.	No correlation between PTH levels and the stimulation index. PTH decreased the stimulation index in hemodialysis patients and increased it in normal subjects at 10 ng/dl.
	Incubated for 64 hours	0.1 to10 ng/ml.	Proliferation assessed with thymidine uptake.	

Tzanno-Martins et al. [44]	Hemodialysis patients lymphocytes with high and low PTH.	PTH measured by immunoradiometry	Stimulation with: Phytohemagglutinin, pokeweed mitogen.	Both group had lower than normal total lymphocytes number. CD4 and CD4/CD8 were higher in high PTH group. Higher secretion of IgM in both group compared to controlled with normal kidney function. Positive correlation between PTH levels and proliferation of lymphoproliferative response of CD4. No difference if the patients were treated by nifedipine and 1,25-hydroxyvitamin D.
			Proliferation assessed with thymidine uptake.	

Ozdemir et al. [45]	54 hemodialysis patients: (1) 20 with PTH< 65 ng/ml. (2) 34 with PTH>300 ng/ml. Only group 2 was treated with VitD replacement.	PTH levels measured by immunometric assay.	N/A	CRP, ferritin concentrations were higher in the group with normal PTH. CD4/CD8 ratio was lower in low PTH group.

**Table 2:** Study of the effects of parathyroidectomy.

	Study design	Assays	Results
Shasha et al. [50]	3 patients with primary hyperparathyroidism pre- and 1 m postparathyroidectomy. Controls: 3 healthy subjects and a male with lipoma.	Stimulation with: Phytohemagglutinin, ConA.	(i) Total T-lymphocytes number were 40% lower that was partially normalized postop. (ii) CD4/CD8 elevated preop normalized postop. (iii) Lymphocytes transformation was inhibited preop it was restored postop.
		Proliferation assessed with thymidine uptake.	

Gaciong et al. [51]	Rats with nephrectomy with or without parathyroidectomy: (1) Intraperitoneal injection of sheep red blood cells. (2) Intramuscular bovine serum albumin. (3) Intramuscular influenza virus vaccine	Dosage of Ig production (IgG and IgM).	(i) The production of IgG was markedly impaired in CKD rats without parathyroidectomy. (ii) The production of IgG was normal in CKD rats with parathyroidectomy. (iii) The production of IgM was lower than normal in rats with CKD with and without parathyroidectomy. (iv) The rats with CKD and without parathyroidectomy had lower IgM levels compared to patients with CKD and parathyroidectomy.

Chervu et al. [52]	5 groups of rats: (1) Normal. (2) With CKD (nephrectomy). (3) CKD + parathyroidectomy (4) CKD + verpamil (5) Normal + verapamil	N/A	(i) Lower ATP content in PMN in CKD versus normal. (ii) Parathyroidectomy and verapamil therapy prevented phagocytosis impairment in CKD. (iii) Verapamil prevented increase intracellular calcium in PMNs.

Kotzmann et al. [53]	12 patients with primary hyperparathyroidism before and 6 m after parathyroidectomy. Cells were cultured for 48 hours	PTH measured by radioimmunoassay.	(i) No change in serum Ig levels after surgery. (ii) Normal distribution pre- and postop for T,Band NK cells. (iii) CD4 elevated and CD8 decreased, CD4/CD8 increased pre- and postop.
		Blood analyzed by flow cytometry.	
		Proliferation determined by thymidine incorporation	

Tzanno-Martins et al. [44]	6 hemodialysis patients with secondary hyperparathyroidism before and 4 m after parathyroidectomy. Cultured for 5 days.	Stimulation with: Phytohemagglutinin.	(i) Lymphoproliferative response increased after parathyroidectomy. (ii) The ability to produce IgG and IgM was increased after parathyroidectomy. (iii) Decrease ability to produce IL-2 was not restored postop.
		Proliferation determined by thymidine incorporation.	

**Table 3:** Study discussing the effect of PTH on B-lymphocytes.

	Study design	PTH	Assays	Results
Alexiewicz et al. [38]	B cells: 21 hemodialysis patients, 37 healthy subjects Cultured for 5 days.	Bovine (1–84) PTH, Purified (1–34) PTH, (1–84) PTH, Inactivated (1–84) PTH.	Stimulation with S. aureus cowan strain I	(i) Lower proliferation potential for B-lymphocytes of hemodialysis patients. (ii) (1–34) and (1–84) PTH produced dose-dependant inhibition of proliferation. (iii) (1–84) inhibitory effect was more than (1–34).
		1 × 10^−7^, 2 × 10^−7^and 4 × 10^−7^ M	Proliferation assessed with thymidine uptake.	

Gaciong et al. [51]	Mononuclears cells: 34 hemodialysis patients, 44 healthy subjects Cultured 8 days.	Bovine (1–84) PTH, bovine (1–34) PTH. PTH was added at the beginning and at day 2.	Stimulation with S. aureus cowan strain I	(i) (1–84) and (1–34) PTH inhibited immunoglobulin production. (ii) At the lower dose of (1–84) PTH had no inhibition of B lymphocytes of ESRD patients. (iii) Inactivated (1–84) had no inhibitory effect. (iv) The inhibition was only observed when PTH was added at the beginning of the culture.
		5 × 10^−7^and 10^−6^ M	Function was assessed by Ig production.	

Jiang et al. [55]	Human B-cell lines (CBL3, SKW6.4, CESS). Incubated for 5 days	Human (1–84) PTH	Stimulation with S. aureus cowan strain I	(i) PTH directly inhibits Ig production by B lymphocytes. (ii) PTH did not affect B-cell growth (iii) IL-4 reduced the inhibitory effect of PTH on Ig production.
		0.01, 0.1, 1, 10, 100 ng/ml.	Proliferation assessed with thymidine uptake.	
